# The microstructure and property of Al–Si alloy improved by the Sc-microalloying and Y_2_O_3_ nano-particles

**DOI:** 10.1080/14686996.2021.1891841

**Published:** 2021-03-10

**Authors:** Jiqiang Chen, Feng Wen, Chao Liu, Weirong Li, Qiongyu Zhou, Wencai Zhu, Yinghui Zhang, Renguo Guan

**Affiliations:** a Faculty of Materials, Metallurgy and Chemistry, Jiangxi University of Science and Technology, Ganzhou, China; b Dongguan Eontec Co., Ltd, Dongguan, China; c School of Materials Science and Energy Engineering, Foshan University, Foshan, China; d Ganzhou Zhanhai New Material Technology Co., Ltd, Ganzhou, China; e Engineering Research Center of Continuous Extrusion, Ministry of Education, Dalian Jiaotong University, Dalian, China

**Keywords:** Al alloy, Sc, Y_2_O_3_ nano-particles, annealing treatment, mechanical property, 103 Composites, 106 Metallic materials, 303 Mechanical / Physical processing, 503 TEM, STEM, SEM

## Abstract

The effect of Sc-microalloying and Y_2_O_3_ nano-particles on the microstructure and mechanical properties of as-cast Al-5.5Si alloy is studied by means of optical microscopy, transmission electron microscopy, hardness test and tensile test. The influence of annealing treatment on the microstructure and properties of the Al-Si alloys is also investigated as well. The results show that the addition of Sc and Y_2_O_3_ nano-particles could significantly improve the mechanical property of the Al-Si alloy. The ultimate tensile strength and yield strength of Al-Si-Sc/Y_2_O_3_ alloy are improved by around 45 and 71%, respectively, when compared to that of the Al-Si alloy. The effect of the nanosized particles (precipitated and added) on strengthening and deformation of Al-Si alloy is analyzed and discussed in detail. The results of annealing treatment indicate that the change in mechanical property of the Al-Si-Sc alloy during annealing treatment is mainly associated with the precipitation of the secondary Si phase.

## Graphical Abstract

**Figure:**
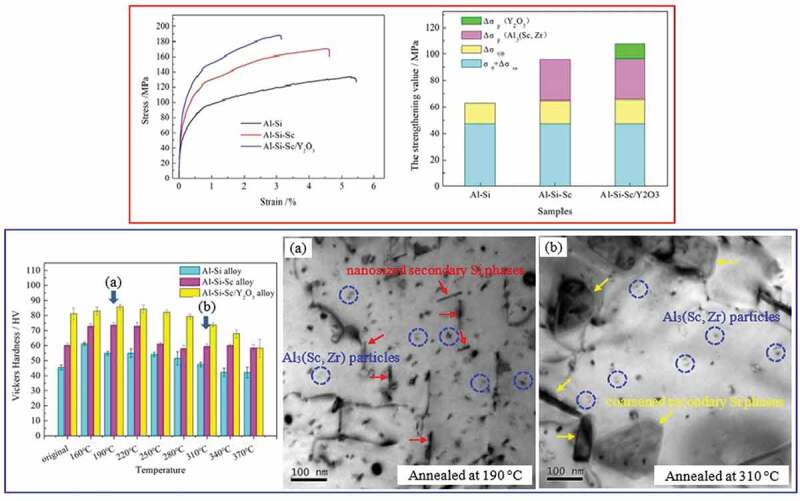

## 1. Introduction

Al–Si alloy is usually used as cast aluminum alloy in many fields such as aerospace and automobile industry, due to its excellent castability, corrosion resistance and good mechanical properties [1,2]. Al–Si alloy is also widely used as welding wire or filler metal due to its low sensitivity to hot cracking and good fluidity when melted. For example, AA 4043 aluminum alloy welding wire is widely used for welding 6000 series wrought aluminum alloys and some cast aluminum alloys. However, the application of Al–Si alloy welding wire is somewhat limited due to the low strength of welding joint. Therefore, effectively improving the strength properties of cast Al–Si alloy used as the filler metal is of great importance.

Several methods could be used to modify the mechanical property of Al alloy. One of the common methods is the microalloying of rare earth, which is very effective in the performance optimization of aluminum alloys. The rare earth Sc is believed to be one of the most effective additives to improve the microstructure and properties of aluminum alloys [3–5]. There are several publications on the modification of Sc addition in the Al-Si-based alloy [6–9], but they are more focused on the modification of the morphology and size of eutectic Si phase or the grain refinement of primary Al phase. Zhang et al. [6] reported that the addition of Sc in as-cast Al–Si alloy could modify the morphology of eutectic Si from plate-like and acicular form to branched and somewhat fibrous one. The average length of the eutectic Si decreases from original 150 to 20 μm when the content of Sc addition is reached 0.4 wt.%. Patakham et al. [7,8] suggested that the addition of Sc could modify the eutectic Si morphology in the Al–Si–Mg foundry alloy, but the grain refinement efficiency of Sc may be less than that of titanium (Ti). In addition, Xu et al. [10] studied the modification of different combinations of Sc and Zr contents (Sc + Zr = 0.5 wt.%) on the Al–7Si–0.65 Mg foundry alloy. The results indicated that the eutectic Si was modified from plate-like morphology to fiber and the microstructure was refined when the content of Sc addition was 0.5 wt.% (without Zr addition).

The modification of nano-particles is another common method that could improve the mechanical property of Al alloys [11–14]. It is reported that the yield strength of A356 alloy was improved by more than 50% with only 2.0 wt.% of nano-sized SiC particles [11]. Kwon et al. [13] prepared aluminum matrix composite materials reinforced with carbon nanotubes (CNT) and SiC nanoparticles, and the Vickers hardness of these composites could be at most eight times higher than that of pure Al bulk. More recently, Lin et al. [14] showed that the laser-printed aluminum reinforced by nanoparticles offered one of the highest specific yield strength and specific Young’s modulus among structural metals. The yield strength of this nanocomposite reached 1000 MPa, and Young’s modulus was approximately 200 GPa, while the elongation exceeded 10%. The Y_2_O_3_ nano-particles are generally used in light-emitting materials [15,16], the modification effect of Y_2_O_3_ nano-particles on metal structural materials is also of interest. Karaka et al. [17] reported that the nano-Y_2_O_3_ dispersed ferritic steel prepared by mechanical alloying and hot isostatic pressing recorded extraordinary compressive strength, Young’s modulus, fracture toughness and hardness. Zaguliaev. et al. [18] studied the element and phase composition, micro-hardness and wear resistance of Al-Si alloy surface layer processed using the method of electron-plasma alloying. The Y_2_O_3_ powder was used to alloying with the surface layer of Al conductor by the method of electric explosion. The results show a significant drop of micro-hardness in layers at depths around 30 μm, and are results of nanocrystallization and Y enrichment of surface layers by electron-plasma alloying. The modification of Y_2_O_3_ nano-particles to the strength of aluminum alloy has not been well studied. It is worth noting that the resource of the rare earth Y is very abundant in the south of Jiangxi province of China, and the price of Y_2_O_3_ nano-particles is not very expensive. In addition, there are also some other methods focused on the changes in the structure and properties of Al-Si alloys, such as super-gravity field assist [19], intense pulse electron beams [20], etc.

In present study, the microalloying method and the modification of nano-particles are both adapted to improve the strength properties of Al–Si alloy that is used as filler metal. Based on the experience of improving the properties of aluminum alloys by co-adding Sc and Zr [10,21], the effect of Sc and Zr on the microstructure and mechanical properties of as cast Al-5.5Si alloy (AA 4043) is studied. As well as the modification effect of Y_2_O_3_ nano-particles addition in Al-Si alloy is studied. The effect of the nanosized particles (precipitated and added) on strengthening and fracturing of Al-Si alloy is analyzed and discussed in detail. Moreover, the influence of annealing treatment on the microstructure and properties of the Al-Si alloys is also investigated. The precipitation of secondary Si phase is first found to play important role in the strengthening of the as cast Al-Si-based alloy during annealing treatment at low temperature. This work aims to provide new insight into the development and performance optimization of Al-Si alloy welding wire.

## 2. Experimental details

### 2.1. The materials preparation

**Figure 1.**
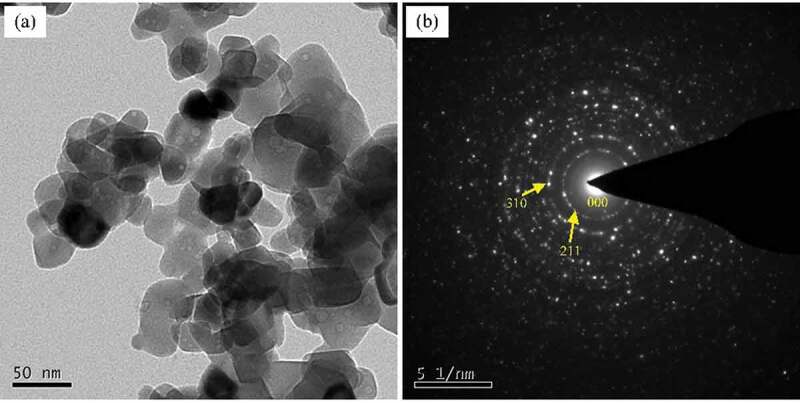
Bright field (BF) TEM image and corresponding SAED pattern of the Y_2_O_3_ nano-particles used in present work: (a) Bright field (BF) TEM image; (b) SAED pattern

AA 4043 alloy, which is the most conventional Al-Si alloy welding wire, is used for modification in present study. The chemical compositions (all in weight percent) of the experimental materials are Al-5.4%Si-0.02%Ti-0.06%Fe (AA 4043, denoted as Al-Si alloy), Al-5.5%Si-0.28%Sc-0.13%Zr-0.07%Fe (denoted as Al-Si-Sc alloy) and Al-5.4%Si-0.27%Sc-0.12%Zr-0.07%Fe-Y_2_O_3_ (denoted as Al-Si-Sc/Y_2_O_3_ alloy), which were determined by the inductively coupled plasma atomic emission spectrometry. All the three alloys were melted in a silicon carbide crucible using a resistance furnace and casted in a preheated iron mold. The detailed preparation procedure was as described in our recent publication [22]. The Y_2_O_3_ nano-particles were provided by the Ganzhou Zhanhai New Material Technology Co., Ltd. A transmission electron microscopy (TEM) image and corresponding selected area electron diffraction (SAED) of the as-received Y_2_O_3_ particles (BCC structure, a = b = c = 1.0604 nm) are shown in Figure 1, which indicated that these particles are nanosized, with a size of the nano-particles about 30 ~ 50 nm. The method of molten salt-assisted incorporation [23] is adapted to add Y_2_O_3_ nano-particles into aluminum. The detailed preparation process is as follows: (i) The Y_2_O_3_ nano-particles (20% in weight percent) and KAlF_4_ powders (80% in weight) mixed sufficiently by using high energy ball milling for over 1 h. (ii) The mixed powders were poured onto the surface of molten Al which was melted at 780°C in a graphite crucible. The weight percent of Y_2_O_3_ nano-particles in Al was designed to be around 6 wt.%, then to about 900°C, and keep stirring the melt for over 20 min. (iii) Taking out the graphite crucible from the furnace, the molten melt was cooled naturally in air, then the composite of aluminum and Y_2_O_3_ nano-particles was obtained. The aluminum incorporated with Y_2_O_3_ nano-particles was used to prepare the Al-Si-Sc/Y_2_O_3_ alloy. The content of Y_2_O_3_ nano-particles in the Al-Si-Sc/Y_2_O_3_ alloy was around 0.8 wt% (~0.43 vol.%), which was estimated by the results of the chemical composition of Y element. The annealing treatment for the three as-cast alloys was designed to be annealing at different temperatures (ranging from 160 to 370°C with 30°C temperature interval) for 1 h.

### 2.2. The mechanical test

The tensile test and hardness test were performed to evaluate the mechanical property of the alloys. The standard flat samples for tensile test were prepared from three as cast alloys, and three parallel samples were tested for each alloy. The gauge length of the test samples was set to be 72 mm determined by 5.65$\sqrt{S_{0}}$ (the S_0_ is the cross-sectional area of the gauge part, which was 20 mm (width)×8 mm (thickness)). A CMT5105 electronic tensile testing machine (Jinan MTS Testing Technology Co., Ltd, China) was employed, performed at room temperature. The testing speed was controlled to be first 2 mm/min and then 10 mm/min after yield stage. The Vickers hardness tests were carried out on a Vickers hardness tester of Type 200HVS-5 (Laizhou Huayin Testing Instrument Co., Ltd, China). The indentation load was 9.8 N (1 kg) and the period of loading is 15 s. A minimum of five hardness values were measured for each sample.

### 2.3. Microstructure characterization

The optical microstructure of the three as cast alloys was analyzed by using a ZEISS Axioskop.A1optical microscope (Carl Zeiss, Germany). The metallographic samples were first mechanically polished, and then etched with regular Keller solutions for 15 to 30 seconds. The intragranular morphology (i.e. second phase, dislocations et al.) of Al matrix were characterized by high-resolution TEM (HRTEM). The TEM samples were first cut from both the as cast alloys (including annealing samples) and the fractured tensile test samples (5 mm away from the fractured location), then mechanically polished to a final thickness of ~80 μm. Subsequently, the foils with diameter of 3 mm were punched and then subjected to twin jet electro-polishing at 20 V using a solution of 90 ml HNO_3_ and 210 ml methanol cooled at −25 to −40°C. The preparation procedures of the TEM samples are similar to the previous studies [22,24–27]. The TEM characterization was performed using FEI TECNAI G2 F20 S-TWIN scanning TEM (STEM) operated at 200 KV.

## 3. Results

### 3.1. The mechanical property

**Figure 2.**
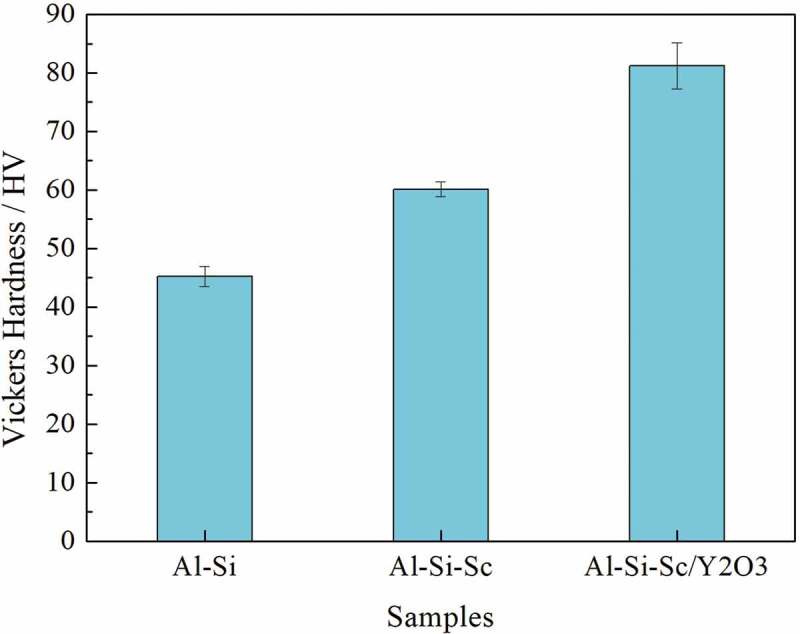
Hardness results of the three alloys in as-cast condition

**Figure 3.**
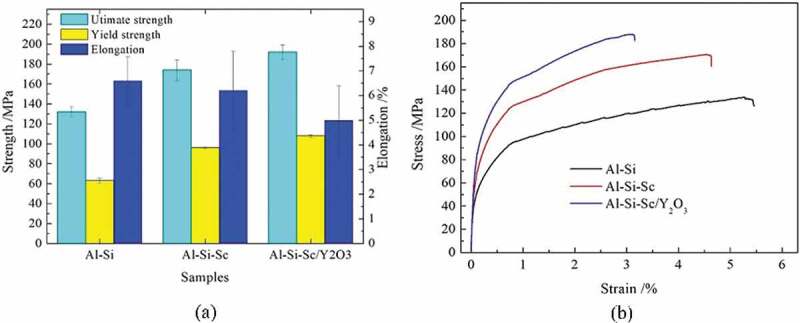
The tensile test results of the three alloys in as-cast condition: (a) comparison of the results of tensile test; (b) the stress–strain curves

Figure 2 shows the Vickers hardness results of the three Al-Si-based alloys in as-cast condition. The hardness value increased from 46HV to 60HV while Sc is added to the Al-Si alloy. Furthermore, the hardness value further increased from 60HV to 80HV when the Y_2_O_3_ nano-particles are added to the Al-Si-Sc alloy. The results indicate that the addition of Sc in the Al-Si alloy improves the hardness by about 33%, and the co-addition of Sc and the Y_2_O_3_ nano-particles to the Al-Si alloy improves the hardness by over 80%. The corresponding tensile mechanical property and stress–strain curves of the three as-cast alloys are shown in Figure 3. The results are consistent with that of the hardness test. The mean ultimate strength, yield strength and elongation of the Al-Si-Sc/Y_2_O_3_ alloy are 192(±7) MPa, 108(±1) MPa and 5.0(±1.4) %, respectively; those of the Al-Si-Sc alloy are 174(±10) MPa, 96(±1) MPa and 6.2(±1.6) %, respectively, while those of the Al-Si alloy are 132(±5) MPa, 63(±2) MPa and 6.6(±1.0) %, respectively. The ultimate tensile strength and the yield strength of the Al-Si-Sc alloy are improved by around 32 and 52%, respectively, with almost no loss in elongation, while those of Al-Si-Sc/Y_2_O_3_ alloy are improved by 45and 71%, respectively, at cost of decrease of 24% in elongation, as compared with those of the Al-Si alloy. It can be found that the addition of Sc and the Y_2_O_3_ nano-particles could significantly improve the mechanical property of the Al-Si alloy.

### 3.2. Microstructure

#### 3.2.1. Optical microstructure

**Figure 4.**
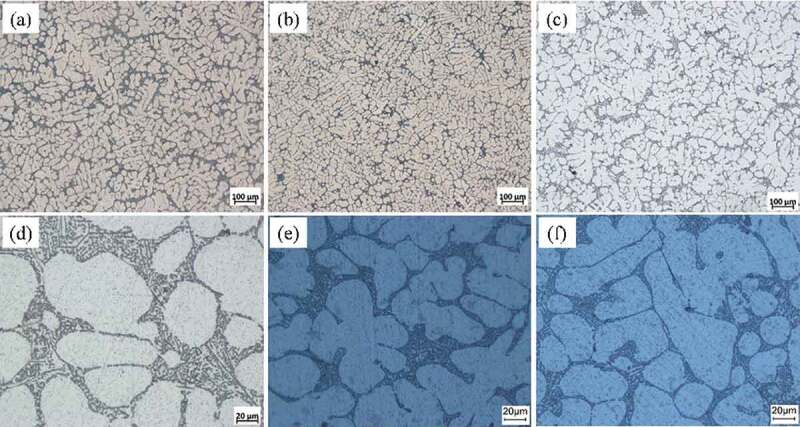
Optical microscopy images of (a, d) Al-Si alloy; (b, e) Al-Si-Sc alloy; (c, f) Al-Si-Sc/Y_2_O_3_ alloy

Figure 4 shows the optical microstructure of the as-cast Al-Si alloy, Al-Si-Sc alloy and Al-Si-Sc/Y_2_O_3_ alloy. All of the three alloys show the typical dendrite structure, but the Al-Si-Sc alloy and Al-Si-Sc/Y_2_O_3_ alloy seem to have finer dendrite grain as compared to the Al-Si alloy. The statistical average grain sizes of the as-cast Al-Si alloy, Al-Si-Sc alloy and Al-Si-Sc/Y_2_O_3_ alloy are 88(±3) μm,70(±5) μm and 65(±2) μm, respectively. The average grain size of the Al-Si-Sc/Y_2_O_3_ alloy is the smallest, and the next is the Al-Si-Sc alloy. It suggests that the addition of Sc can refine the grain size on the basis of the commercial Al-Si alloy (i.e. AA 4043 alloy). In addition, the eutectic Si phases of the three alloys are mainly concentrated in between dendrites of the Al matrix. The morphology of the eutectic Si phases in the three alloys is fine and some of them seem to fibrous, as shown in Figure 4(d,e,f). The size of the eutectic Si phase in Al-Si-Sc alloy and Al-Si-Sc/Y_2_O_3_ alloy is smaller as compared to that of the eutectic Si phase in Al-Si alloy. The effect of the Sc addition on the eutectic Si phase in Al-Si alloy has been discussed in detail in our previous work [22], the addition of Sc into the Al-Si alloy could also increase the stacking faults in the Si phase, and further modify the Si phase based on the mechanism of impurity induced twinning.

#### 3.2.2. TEM microstructure

**Figure 5.**
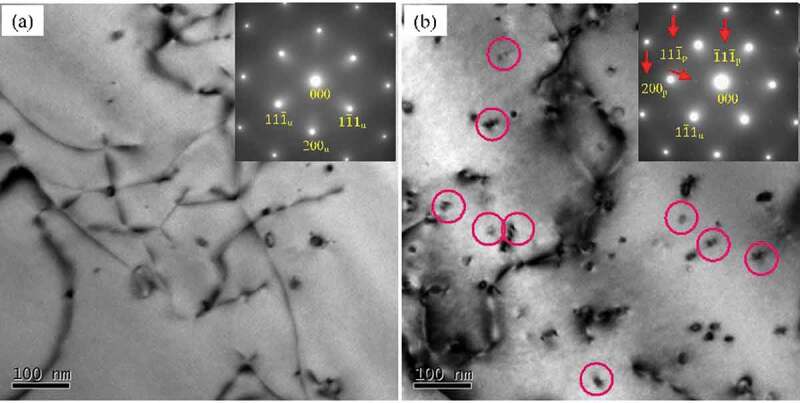
BF TEM images and corresponding SAED patterns of as-cast alloys: (a) Al-Si alloy; (b) Al-Si-Sc alloy

Figure 5 shows the BF TEM images and corresponding SAED patterns of as-cast Al-Si alloy and Al-Si-Sc alloy. It can be found that there are few precipitates in the matrix of Al-Si alloy, while there are numbers of nanosized precipitates in the matrix of Al-Si-Sc alloy. The morphology and the SAED patterns indicate that these nano precipitates are the Al_3_(Sc, Zr) particles (indicated by the red circle in Figure 5(b)), which are the regular second phase in Al-Sc-Zr alloy system [28]. These Al_3_(Sc, Zr) particles greatly contribute to the strength property of Al-based alloy due to the precipitation strengthening mechanism [5,29], and is the other main reason for the strength difference between the as-cast Al-Si alloy and Al-Si-Sc alloy.

**Figure 6.**
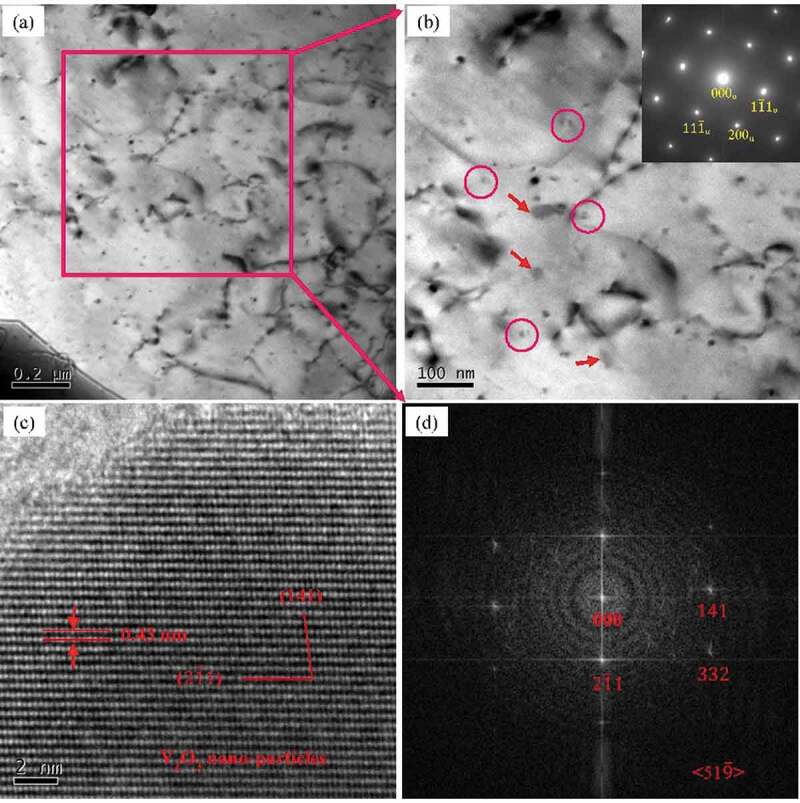
BF TEM images and corresponding SAED patterns of as-cast Al-Si-Sc/Y_2_O_3_ alloy: (a) (b) BF TEM images; (c) HRTEM images of Y_2_O_3_ nano-particles; (d) the FFT image corresponding to (c)

Accordingly, the TEM images and corresponding SAED patterns of as-cast Al-Si-Sc alloy with Y_2_O_3_ nano-particles are shown in Figure 6. Similar to the as-cast Al-Si-Sc alloy, there are also numbers of Al_3_(Sc, Zr) particles (as indicated by the red circle in Figure 6(b)). Furthermore, the Y_2_O_3_ nano-particles with dimension of 30–50 nm are evident in the Al matrix as well, which are indicated by the arrows in Figure 6(b). The corresponding HRTEM images of the Y_2_O_3_ nano-particles are presented in Figure 6(c). Figure 6(d) is the fast Fourier transform (FFT) image corresponding to Figure 6(c) and shows the typical pattern of BCC crystal structure, which is consistent with the structure of Y_2_O_3_ (BCC, a = b = c = 1.0604 nm). It is reasonable to consider that the nanosized Al_3_(Sc, Zr) particles and Y_2_O_3_ particles play critical role in strengthening for the Al-Si-Sc/Y_2_O_3_ alloy. The contribution of the nanosized Al_3_(Sc, Zr) particles and Y_2_O_3_ particles to the strength of the Al-Si-Sc/Y_2_O_3_ alloy would be discussed in the next section in detail.

### 3.3. The microstructure and property after annealing

**Figure 7.**
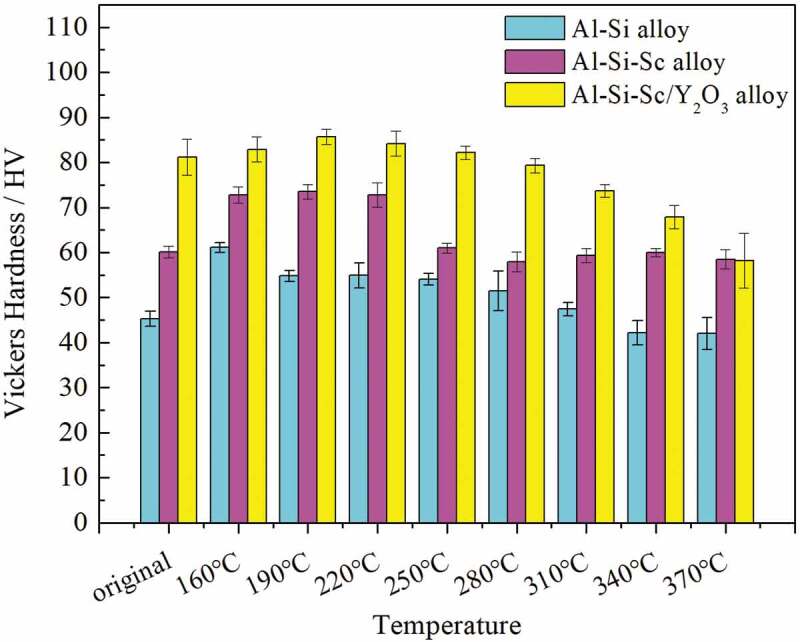
The hardness evolution of the Al-Si-based alloys after annealing at the different temperature for 1 hour

The hardness evolution of three Al-Si-based alloys after annealing at the different temperatures is shown in Figure 7. The result demonstrates that the annealing treatment has significant effect on the property of the three alloys. The hardness of the three alloys after annealing present similar trends: the hardness increase (i.e. higher than that of the original temper) at the relatively low annealing temperatures (160– 250°C), and the hardness decrease (i.e. lower than that of the original temper) while the annealing temperature exceeds 280°C. The highest hardness is achieved when the annealing temperature is around 190°C for the Al-Si-Sc alloy and Al-Si-Sc/Y_2_O_3_ alloy, or around 160°C for the Al-Si alloy. This hardness change is generally associated with the second phase or precipitates in the matrix. It is worth noting that the effect of annealing treatment on the property in Al-Si-Sc alloy is significantly different from those in other Al-Sc alloy systems, such as Al-Mg-Sc alloy [30,31]. As reported, the effect of annealing treatment on the Al-Sc alloy is mainly associated with the precipitation of secondary Al_3_Sc or Al_3_(Sc, Zr) particles, and the optimum temperature for the precipitation of secondary Al_3_Sc or Al_3_(Sc, Zr) particles is generally considered to be around 300°C [30,31]. Unfortunately, the hardness is decreasing while the annealing treatment reaches or exceeds 300°C according to the result shown in Figure 7 in present work. It suggests that the precipitation of secondary Al_3_(Sc, Zr) particles may not play a dominant role in the Al-Si-Sc alloy after annealing, or the hardness increasing of the alloys after annealing have little to do with the precipitation of secondary Al_3_(Sc, Zr) particles. In addition, the results shown in Figure 7 indicate that the heat-resistant of Al-Si-Sc alloy and Al-Si-Sc/Y_2_O_3_ alloy is higher than that of the Al-Si alloy. This result may be associated with the existing Al_3_(Sc, Zr) particles, which were reported to have highly resistant in high temperature [5,29].

**Figure 8.**
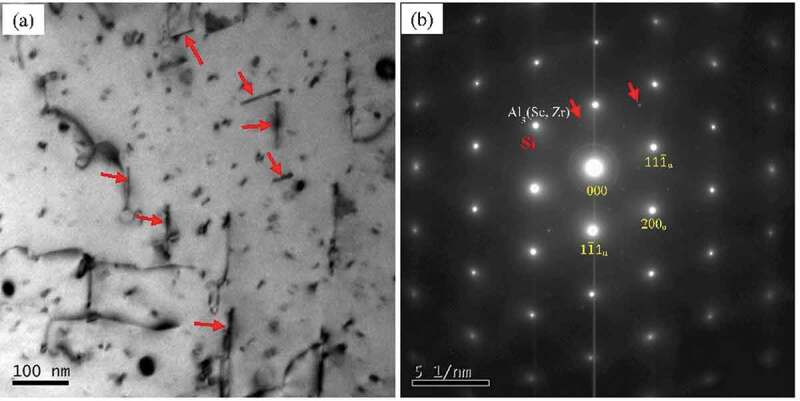
(a) BF TEM images of the Al-Si-Sc alloy after annealing at 190°C for 1 hour, (b) The SAED pattern corresponding to (a), the white arrows indicating the signal of Al_3_(Sc, Zr) particles while the red arrows indicating the signal of secondary Si phase

**Figure 9.**
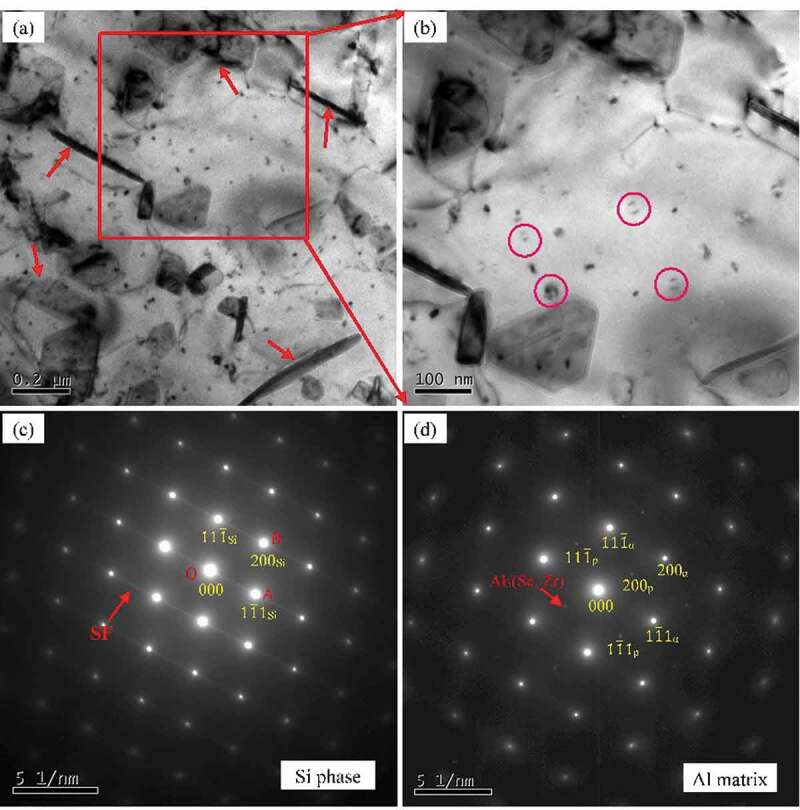
(a) BF TEM images of the Al-Si-Sc alloy after annealing at 310°C for 1 hour; (b) A local zoom of (a); (c) The SAED pattern of the coarse phase in Al matrix, the streaks indicate the signal of the stacking faults (SF); (d) The SAED pattern of the Al matrix, the signal of the Al_3_(Sc, Zr) particles is evident

In order to investigate the microstructure evolution of the alloys after annealing, Al-Si-Sc alloy is chosen as the typical sample to study the TEM microstructure. Figure 8 shows the TEM images and corresponding SAED pattern of the Al-Si-Sc alloy after annealing at 190°C for 1 h. In addition to the Al_3_(Sc, Zr) particles, there are numbers of needle-shaped second phase with length of 50 nm~100 nm in Al matrix, which are indicated by the red arrows in Figure 8(a)). The SAED pattern (indicated by red arrows in Figure 8(b)) suggests that these nanosized needle-shaped second phase are the secondary Si phase. Accordingly, the TEM images and corresponding SAED pattern of the Al-Si-Sc alloy after annealing at 310°C for 1 h are shown in Figure 9. In addition to the Al_3_(Sc, Zr) particles (as indicated by the circles in Figure 9(b)), there are numbers of coarse second phases with the dimension of 200 **~ **500 nm in the matrix, as indicated by the arrows in Figure 9(a). The SAED pattern of these coarse second phases is shown in Figure 9(c), and the SAED pattern of Al matrix is also presented in Figure 9(d) for comparison. Figure 9(c) shows that the coarse second phase has similar cubic structure to Al matrix, but has different d-spacing as well as characteristic stacking faults (SF) in SAED pattern. Based on the results of measurement and calculation, the reciprocal of the length of OA (1/L_OA_ = 0.322 nm) and OB (1/L_OB_ = 0.278 nm) are very close to the values of *d*_111_ (0.313 nm) and *d*_200_ (0.272 nm) of Si phase (a = 0.5431 nm), respectively. It could confirm that these coarse second phases are also the Si phase.

Comparing the microstructure of Al-Si-Sc alloy sample in as-cast temper (Figure 5(b)) to that of the samples after annealing (Figures 8 and Figures 9(b)), it can be found that there are not pronounced changes in the size or number density of Al_3_(Sc, Zr) particles. This result confirms that the precipitation of Al_3_(Sc, Zr) particles may not play a dominant role in the property changes of the alloy after annealing. Furthermore, comparing the microstructure of Al-Si-Sc alloy between Figures 8 and Figures9, the main difference of the microstructure under two different post-annealing temperatures is the dimension and morphology of the secondary Si phase. At the relatively low post-annealing temperature, the nanosized secondary Si phases are precipitated in the Al matrix, and they could improve the strength property of the Al-Si-Sc alloy. This may be the main reason for the results that the hardness is increasing in the alloy after annealing at low temperatures as shown in Figure 7. On the other hand, the secondary Si phase would be significantly coarsened when the post-annealing temperature is relatively high (i.e. 310°C). The coarsened secondary Si phase affects the effect of strengthening, and lead to the decreasing of the hardness of Al-Si-Sc alloy under the relatively high post-annealing temperatures.

## 4. Discussion

### 4.1. The effect of the nanosized particles on strengthening of Al-Si alloy

The mechanical property results demonstrate that the addition of Sc and the Y_2_O_3_ nano-particles could significantly promote the strength of the Al-Si alloy. The contribution of Sc and Y_2_O_3_ nano-particles in the strengthening of Al-Si-based alloy is of interest and would be discussed in detail. Four strengthening mechanisms were generally introduced to explain the strengthening of the Al alloy system [21,29,32,33], they are solid solution strengthening ($\sigma_{ss}$), dislocation strengthening ($\sigma_{d}$), grain boundary strengthening ($\sigma_{GB}$) and precipitation strengthening ($\sigma_{p}$), respectively. The strength of the Al alloy could be represented by $\sigma_{s}$=$\sigma_{0}$+$\sigma_{ss}$+$\sigma_{d}$+$\sigma_{GB}$+$\sigma_{p}$, if we assume that these four strengthening mechanisms act independently and thus have additive contributions. For the as-cast alloy, the dislocation strengthening ($\sigma_{d}$) could be out of consideration. The solid solution strengthening ($\sigma_{ss}$) is associated with the concentration of the solute atoms in the matrix. Since the annealing treatment of Al-Si-Sc alloy has little effect on the precipitation of the Al_3_(Sc, Zr) particles, the concentration of Sc atoms in the matrix is estimated to be very limited. For this reason, the value of ($\sigma_{0}$+$\sigma_{ss}$) is considered to be almost equal for the three alloys in present work. The strength difference of the three alloys is therefore speculated to be originated from the grain boundary strengthening ($\Delta \sigma_{GB}$) and precipitation strengthening ($\Delta \sigma_{p}$). The estimated values of the contribution of each strengthening approach are as shown in Table 1, and the corresponding details are as following:

**Table 1.** The estimated values of the contribution of each strengthening approach

Alloys	$\sigma_{0}$+$\sigma_{ss}$/MPa (balance^1^)	$\Delta \sigma_{GB}$/MPa (estimate^2^)	$\Delta \sigma_{p}$/MPa				The yield strength $\sigma_{s}$/MPa	
			Al_3_(Sc, Zr)		Y_2_O_3_			
			Balance^1^	Estimate^2^	Balance^1^	Estimate^2^	Actual value	Estimated value
Al-Si	47	16	/	/	/	/	63	/
Al-Si-Sc	47	18	31	36	/	/	96	101
Al-Si-Sc/Y_2_O_3_	47	19	31	36	11	15	108	117

1. The values of ‘balance’ are balanced from the actual value; 2. The values of ‘estimate’ are estimated by the strengthening model.

(i) The value of grain boundary strengthening ($\Delta \sigma_{GB}$): The contribution of grain boundary strengthening ($\sigma_{GB}$) is generally estimated by the well-known Hall–Petch relationship:

(1) $$\sigma_{GB}=\sigma_{0}+kd^{-1/2}$$

where $\sigma_{0}$ is the intrinsic resistance of the lattice to dislocation motion, *k* is the constant and *d* is the average grain size of the Al alloy. Since $\sigma_{0}$ is equal for the three Al-Si-based alloys, $\Delta \sigma_{GB}=kd^{-1/2}$ is employed to isolate the grain size dependence in present work. Based on the previous studies, the constant of *k* was reported to vary from 0.15 to 0.26 MPam^1/2^ in Al-Mg alloy [34,35]. If *k* = 0.15 MPam^1/2^ is taken in present study, the contribution of grain boundary strengthening ($\Delta \sigma_{GB}$) to the strength of the three Al-Si alloys are estimated to be the values as shown in Table 1, based on the results of the average grain size.

(ii) The value of ($\sigma_{0}$+$\Delta \sigma_{ss}$): Since the precipitation strengthening could be almost out of consideration in the Al-Si alloy, the strength of the Al-Si alloy would be composed of the ($\sigma_{0}$+$\sigma_{ss}$) and the grain boundary strengthening ($\Delta \sigma_{GB}$). Then, the grain boundary strengthening ($\Delta \sigma_{GB}$) value is deducted from the measured yield strength of Al-Si alloy (63MPa, Figure 3), the value of ($\sigma_{0}$+Δ$\sigma_{ss}$) is, therefore, 47MPa, which is also available to the Al-Si-Sc alloy and Al-Si-Sc/Y_2_O_3_ alloy.

(iii) The value of precipitation strengthening ($\Delta \sigma_{p}$) due to Al_3_(Sc, Zr) particles: The strengthening contribution of Al_3_(Sc, Zr) particles could be balanced from the actual yield strength. For the Al-Si-Sc alloy, the value of ($\sigma_{0}$+$\sigma_{ss}$) and the grain boundary strengthening ($\Delta \sigma_{GB}$) value are deducted from the measured yield strength of Al-Si-Sc alloy (96MPa, Figure 3), the value of precipitation strengthening ($\Delta \sigma_{p}$) due to Al_3_(Sc, Zr) particles is therefore obtained to be 31.1MPa. The strengthening contribution of Al_3_(Sc, Zr) particles could also be estimated by the strengthening model [29,32]. Strengthening of the alloy from the fine Al_3_(Sc, Zr) particles is generally considered to be associated with particle shearing or with particle bowing mechanisms. For the small size precipitates like Al_3_(Sc, Zr) particles (~10 nm), the first mechanism (particle shearing) is considered to play the dominant role, whereas the second mechanism (particle bowing) is more suitable for coarser particles [29]. In this case, the strengthening contribution of Al_3_(Sc, Zr) particles is believed to result primarily from the formation of an anti-phase boundary (APB) within the sheared particle. The strengthening of Al_3_(Sc, Zr) particles due to the APB mechanism may be predicted using Equation (2) [29],

(2) $$\sigma_{p}=\left(3.1\right)\frac{\gamma^{3/2}}{b^{2}}{\left(\frac{rf}{G}\right)}^{1/2}$$

Where γ is the energy required to form the APB, *r* is the radius of the particles, *f* is the volume fraction of particles, *G* is the shear modulus (*G* is approximately 26 GPa for Al) and *b* is the Burgers vector of dislocations (*b* = 0.286 nm for Al). Based on the TEM images, the diameter of Al_3_(Sc, Zr) particles is around 10 nm, and the volume fraction (*f*) is estimated to be approximately 0.007. If the APB energy γ = 0.185 J/m^2^ (as recommended by Kendig and Maricle [29]) is adopted, the Δσ_p_ introduced by Al_3_(Sc, Zr) particles is estimated to be ~36 MPa, which is very close to the balanced value (31 MPa) as shown in Table 1.

(iv) The value of strengthening ($\Delta \sigma_{Orowan}$) due to Y_2_O_3_ nano-particles: The strengthening contribution of Y_2_O_3_ nano-particles could be balanced from the actual yield strength. For the Al-Si-Sc/Y_2_O_3_ alloy, the value of ($\sigma_{0}$+$\sigma_{ss}$), grain boundary strengthening ($\Delta \sigma_{GB}$) and the precipitation strengthening ($\Delta \sigma_{p}$) due to Al_3_(Sc, Zr) particles are all deducted from the measured yield strength of Al-Si-Sc/Y_2_O_3_ alloy (108 MPa, Figure 3), the value of strengthening contribution ($\Delta \sigma_{Orowan}$) due to Y_2_O_3_ nano-particles is therefore obtained to be ~11 MPa. The strengthening contribution of Y_2_O_3_ nano-particles could also be estimated by the strengthening model. If the Orowan strengthening [36] is introduced to evaluate the strengthening of the Y_2_O_3_ nano-particles, the strengthening due to the Orowan mechanism may be predicted using Equation (3) [36], which was once used to estimate the strengthening contribution of TiC nanoparticles in Al [14].

(3) $$\sigma_{Orowan}=0.13Gb*\ln\left(\frac{r}{b}\right)/\lambda$$

Where *G* is the shear modulus (*G* is approximately 26 GPa for Al), *b* is the Burgers vector of dislocations (*b* = 0.286 nm for Al), *r* is the particle radius and *λ* is the inter-particle spacing, which could be estimated by Equation (4) [14,36]:

(4) $$\lambda \approx d_{p}*\left[{\left(1/2V_{p}\right)}^{1/3}-1\right]$$

Where *d_p_* is the particle diameter and *V_p_* is the volume fraction of nanoparticles. The particle size of Y_2_O_3_ nano-particles is estimated by the TEM images shown in Figure 1. The value of the average radius is 40 nm (Figure 1), and the *V_p_* is about 0.43 vol.%. Then, the Δσ_Orowan_ introduced by Y_2_O_3_ nano-particles is determined to be ~15 MPa, which is slightly higher than the balance value (11 MPa) as shown in Table 1, but it is still acceptable.

**Figure 10.**
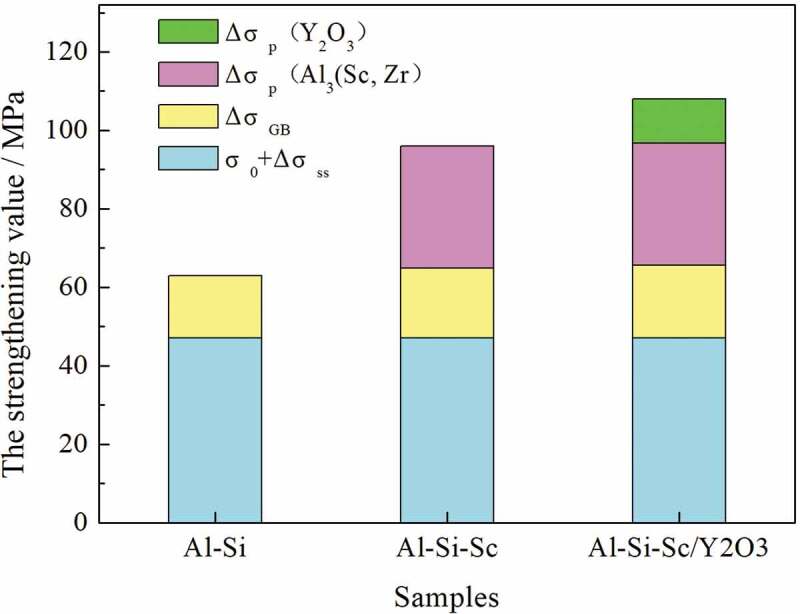
The contribution of each strengthening mechanism to the practical yield strength of the three alloys

The estimated values of the contribution of each strengthening approach in the three alloys could be illustrated as shown in Table 1, and the corresponding contributions to the practical yield strength of the three alloys is also presented in Figure 10. It can be found that the precipitation strengthening of the Al_3_(Sc, Zr) particles is the most remarkable. Meanwhile, the addition of Y_2_O_3_ nano-particles has also provided strengthening to the Al-Si alloy, although the strengthening value is relatively low, which should be attributed to the relatively less contents of Y_2_O_3_ nano-particles.

**Figure 11.**
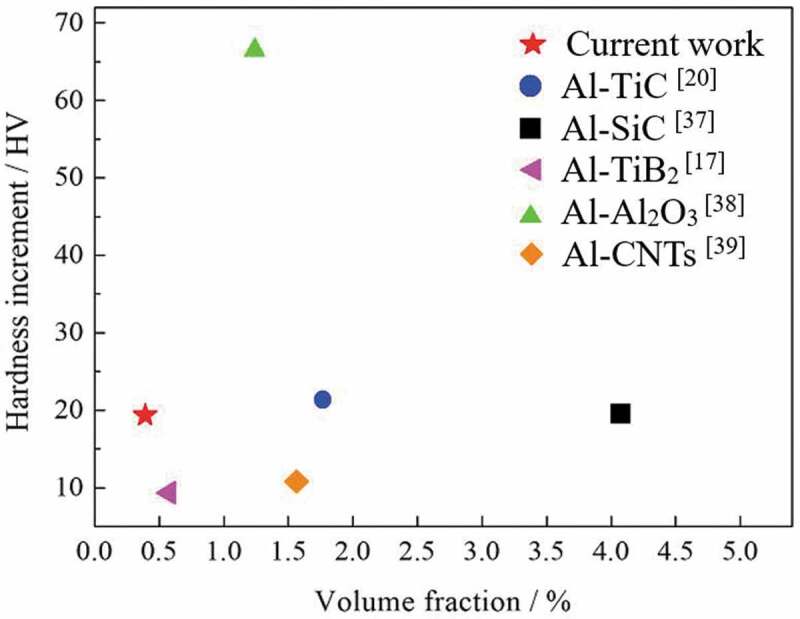
Comparison of hardness increment (HV_nc_−HV_ma_) of Al matrix nanocomposite, where the subscript ‘nc’ represents nanocomposite, and ‘ma’ represents matrix alloy. CNTs is the carbon nanotubes. ‘HV_nc_−HV_ma_’ represents the hardness values of the nanocomposite minus that of the Al matrix. The volume fraction is the content of the added nanoparticles (or nanotubes)

To better evaluated the strengthening effect of the Y_2_O_3_ nano-particles, the hardness increment (HV_nc_−HV_ma_) is introduced in present work, where HV_nc_ represents the hardness values of the nanocomposite and HV_ma_ represents the hardness values of the Al matrix. The hardness increment generated by the Y_2_O_3_ nano-particles are compared with that from the reported researches of Aluminum modified by low content of nanoparticles [13,23,37–39], and the result is shown in Figure 11. Although the result is influenced by different preparation methods or Al matrix, the modification effect of Y_2_O_3_ nano-particles in strengthening of Al alloy is remarkable, and has good application potential in aluminum alloy.

### 4.2. The effect of the nanosized particles on the deformation of Al-Si alloy during tensile test

**Figure 12.**
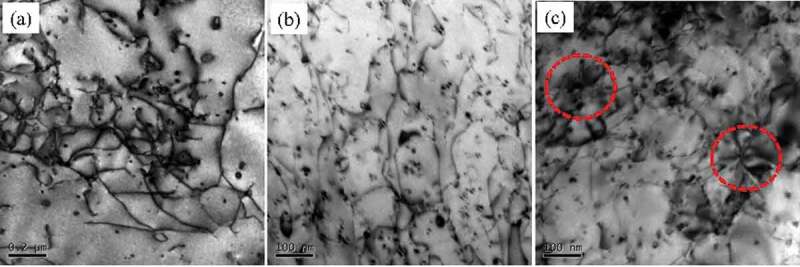
TEM images of the samples that experienced tensile test: (a) Al-Si alloy; (b) Al-Si-Sc alloy; (c) Al-Si-Sc/Y_2_O_3_ alloy. All the images are taken along <011> zone axis

The TEM microstructure of the samples that experienced tensile test is demonstrated to evaluate the effect of the precipitates and nano-particles on the plastic deformation during the tensile test, as shown in Figure 12. It can be found that the deformation in Al-Si alloy is not very uniform and the dislocations are entangled together in a small area. Unlike the Al-Si alloy, the deformation in the Al-Si-Sc alloy is relatively uniform, which should be associated with the uniformly dispersed distribution of Al_3_(Sc, Zr) particles. The existence of these uniformly dispersed Al_3_(Sc, Zr) particles can give full play to the plasticity of the Al-Si-Sc alloy, so as to obtain the elongation close to that of Al Si alloy (Figure 3). For the Al-Si-Sc/Y_2_O_3_ alloy, due to the local concentration of dislocations, it can be considered that the deformation of this alloy is relatively uneven. The stress concentration (as indicated by the circle in Figure 12(c)) is evident in the sample that experienced tensile test. This stress concentration is speculated to be induced by the Y_2_O_3_ nano-particles, because the Y_2_O_3_ nano-particles have larger size than the Al_3_(Sc, Zr) particles and are generally not coherent with the Al matrix. The uneven deformation and the stress concentration lead to the loss of plasticity in Al-Si-Sc/Y_2_O_3_ alloy when compared with the Al-Si alloy and Al-Si-Sc alloy.

### 4.3. The effect of annealing treatment on microstructure and mechanical property

Generally, the precipitation of secondary Al_3_(Sc, Zr) particles plays the dominant role in the property of the Al-Sc-Zr alloy system after annealing [31,32]. However, the experimental results in present work suggest that the effect of annealing treatment on the microstructure and property of the Al-Si-Sc alloy is mainly associated with the precipitation of the secondary Si phase, and has little to do with the precipitation of secondary Al_3_(Sc, Zr) particles. Obviously, the effect of annealing treatment in Al-Si-Sc alloy is unexpectedly different from the other Al-Sc alloys, such as Al-Mg-Sc alloy, which could be further strengthened by the dispersed secondary nanosized Al_3_(Sc, Zr) particles when the annealing treatment is conducted at temperatures above 300°C [31,32]. This difference may be caused by the different solid solubility of Sc in Al-Si-based alloy and the Al-Mg-based alloy may take the main responsibility. The exact reason that the solid solubility of Sc is so different in Al-Si and Al-Mg alloy system is not clear, but it should be associated with Si element, considering the existing form of Sc in Al-Si-based alloy. In addition to the form of solid solution in Al matrix, there are three forms of Sc in the Al-Si-based alloy based on reported researches: (i) The form of Al_3_(Sc, Zr) particles in present work; (ii) The form of AlSi_2_Sc_2_ phase reported by Pandee et al. [40]; (iii) In the eutectic Si phase that plays the role of metamorphism, based on impurity-induced twinning (IIT) mechanism [8,22,41].

Moreover, the secondary Si phases were precipitated in the matrix during the annealing treatment, it suggests that the solid solubility of Si is relatively high in the Al matrix. According to the Al-Si binary phase diagram [42], the Si has little solubility in the Al matrix at the room temperature, but the maximum solubility of Si could reach 1.65 wt% in the Al matrix at high temperature. Under the condition of non-equilibrium solidification, it is very reasonable that a certain amount of Si atoms is dissolved in aluminum matrix. The experimental results shown in Figure 7 suggest that the precipitation of the secondary Si phase is very sensitive to the annealing temperature. Under the relatively low temperature (such as 190°C), the nanosized secondary Si phase with needle shape is precipitated (Figure 8). However, the secondary Si phase would grow fast when the post-annealing temperature exceeds 250°C, and the size of the secondary Si phase could reach 200 ~ 500 nm (Figure 9(a)) when the post-annealing temperature is as high as 310°C. Thus, if higher strength of the Al-Si alloy is expected, it is better to conduct the annealing treatment at a low temperature for longer time.

## 5. Conclusions

The effect of Sc-microalloying and the Y_2_O_3_ nano-particles on the microstructure and mechanical properties of as-cast Al-5.5Si alloy (AA 4043) was studied. The influence of annealing treatment on the microstructure and properties of the Al-Si alloys was investigated as well. The main conclusions could be summarized as follows:

(1) The addition of Sc and the Y_2_O_3_ nano-particles could significantly improve the mechanical property of the Al-Si alloy. The mean ultimate strength, yield strength and elongation of the Al-Si-Sc/Y_2_O_3_ alloy are 192(±7) MPa, 108(±1) MPa and 5.0(±1.4)%, respectively; those of the Al-Si-Sc alloy are 174(±10) MPa, 96(±1) MPa and 6.2(±1.6)%, respectively, while those of the Al-Si alloy are 132(±5) MPa, 63(±2) MPa and 6.6(±1.0)%, respectively. The ultimate tensile strength and the yield strength of the Al-Si-Sc alloy were improved by around 32 and 52%, respectively, with almost no loss in elongation, while those of Al-Si-Sc/Y2O3 alloy are improved by 45 and 71%, respectively, at cost of decrement of 24% in elongation, as compared with those of the Al-Si alloy.

(2) The nanosized Al_3_(Sc, Zr) particles take the main responsibility for the strengthening in Al-Si-Sc alloy and Al-Si-Sc/Y_2_O_3_ alloy, while the Y_2_O_3_ nano-particles show great potential in the strengthening of Al alloy.

(3) The hardness of the three Al-Si-based alloys increases first, and then decreases with increasing the annealing temperature. The highest hardness is achieved when the post-annealing temperature is set within 160 to 190°C.

(4) The effect of the annealing treatment on the mechanical property of the Al-Si-(Sc) alloys is mainly associated with the precipitation of secondary Si phase, and has little to do with the precipitation of secondary Al_3_(Sc, Zr) particles. The nanosized secondary Si phases are precipitated at low annealing temperatures, while the secondary Si phases grow rapidly and coarsen at relatively high annealing temperatures.

## Data Availability

The raw/processed data required to reproduce these findings cannot be shared at this time as the data also form part of an ongoing study.
